# Developing a predictive model for spinal shock in dogs with spinal cord injury

**DOI:** 10.1111/jvim.16352

**Published:** 2022-01-10

**Authors:** Rebecca McBride, Elizabeth Parker, Rebecca B. Garabed, Natasha J. Olby, Andrea Tipold, Veronika Maria Stein, Nicolas Granger, Ashley C. Hechler, Page E. Yaxley, Sarah A. Moore, CANSORT‐SCI study investigators

**Affiliations:** ^1^ Department of Veterinary Clinical Sciences The Ohio State University College of Veterinary Medicine Columbus Ohio USA; ^2^ Department of Veterinary Preventive Medicine The Ohio State University Columbus Ohio USA; ^3^ Department of Clinical Sciences College of Veterinary Medicine, North Carolina State University Raleigh North Carolina USA; ^4^ Department of Small Animal Medicine and Surgery University of Veterinary Medicine Hannover Germany; ^5^ Department of Clinical Veterinary Sciences, Vetsuisse Faculty University of Bern Bern Switzerland; ^6^ Department of Small Animal Clinical Sciences, School of Veterinary Sciences University of Bristol Bristol United Kingdom

**Keywords:** fibrocartilaginous infarct, herniation, intervertebral disc disease, neurology, spinal cord disease, spinal shock

## Abstract

**Background:**

Reduced pelvic limb reflexes in dogs with spinal cord injury typically suggests a lesion of the L4‐S3 spinal cord segments. However, pelvic limb reflexes might also be reduced in dogs with a T3‐L3 myelopathy and concurrent spinal shock.

**Hypothesis/Objectives:**

We hypothesized that statistical models could be used to identify clinical variables associated with spinal shock in dogs with spinal cord injuries.

**Animals:**

Cohort of 59 dogs with T3‐L3 myelopathies and spinal shock and 13 dogs with L4‐S3 myelopathies.

**Methods:**

Data used for this study were prospectively entered by partner institutions into the International Canine Spinal Cord Injury observational registry between October 2016 and July 2019. Univariable logistic regression analyses were performed to assess the association between independent variables and the presence of spinal shock. Independent variables were selected for inclusion in a multivariable logistic regression model if they had a significant effect (*P* ≤ .1) on the odds of spinal shock in univariable logistic regression.

**Results:**

The final multivariable model included the natural log of weight (kg), the natural log of duration of clinical signs (hours), severity (paresis vs paraplegia), and pelvic limb tone (normal vs decreased/absent). The odds of spinal shock decreased with increasing weight (odds ratio [OR] = 0.28, *P* = .09; confidence interval [CI] 0.07‐1.2), increasing duration (OR = 0.44, *P* = .02; CI 0.21‐0.9), decreased pelvic limb tone (OR = 0.04, *P* = .003; CI 0.01‐0.36), and increased in the presence of paraplegia (OR = 7.87, *P* = .04; CI 1.1‐56.62).

**Conclusions and Clinical Importance:**

A formula, as developed by the present study and after external validation, could be useful for assisting clinicians in determining the likelihood of spinal shock in various clinical scenarios and aid in diagnostic planning.

## 1 INTRODUCTION

Spinal cord injury is common in dogs, with the thoracolumbar spinal cord segments being the most common sites for injury.^1^ Dogs that are presented with paraparesis or paraplegia, where the thoracic limbs are neurologically normal, most often have lesions involving either the T3‐L3 or L4‐S3 spinal cord segments.^1^, ^2^ Typically, reduced pelvic limb reflexes are suggestive of a lesion of the L4‐S3 spinal cord segments; however, these might also be reduced in dogs with a T3‐L3 myelopathy and concurrent spinal shock.^3^, ^4^, ^5^

Spinal shock is characterized by reduced segmental spinal reflexes and muscle tone caudal to an injury to the spinal cord, despite local reflex arcs remaining physically intact.^1^, ^3^, ^4^, ^5^, ^6^ For dogs with a T3‐L3 myelopathy, this presents as transiently reduced segmental spinal reflexes in the pelvic limbs, giving a false appearance of a lower motor neuron lesion. The condition is thought to be caused by acute injury‐associated interruption in descending, primary faciliatory, motor tracts and transient localized changes in the reflex centers of the spinal cord.^3^, ^5^, ^6^, ^7^, ^8^, ^9^, ^10^, ^11^, ^12^, ^13^, ^14^, ^15^, ^16^, ^17^, ^18^, ^19^, ^20^, ^21^, ^22^, ^23^, ^24^, ^25^, ^26^, ^27^, ^28^, ^29^, ^30^, ^31^, ^32^, ^33^, ^34^, ^35^, ^36^, ^37^, ^38^, ^39^, ^40^, ^41^, ^42^, ^43^, ^44^, ^45^, ^46^, ^47^, ^48^, ^49^, ^50^, ^51^, ^52^ Currently, prioritization of spinal shock over other causes of reduced pelvic limb reflexes and other lower motor neuron signs is solely based on the clinician's intuition and is highly influenced by training and experience. Specifically, the clinical presentation of spinal shock can lead to clinical confusion in dogs with T3‐L3 myelopathies, and might cause the clinician to improperly localize the lesion, exclude the affected area from diagnostic imaging or to prioritize differentials with more guarded prognoses than intervertebral disc disease, such as multifocal inflammatory myelopathies or progressive myelomalacia.

Spinal shock occurs in about 50% of people with acute spinal cord injury, making neuroanatomic localization and assessment of the severity of spinal cord injury a common problem for physicians.^12^, ^13^, ^26^, ^29^, ^32^, ^35^, ^36^, ^37^, ^52^, ^53^ While spinal shock is commonly described in people, and often clinically appreciated in veterinary medicine, only 2 studies have described the condition in dogs with clinical spinal cord injury.^3^, ^4^ Developing a better understanding of what clinical criteria are related to T3‐L3 myelopathy with concurrent spinal shock vs L4‐S3 myelopathy could help guide clinical decision making.

As spinal shock appears to be an underreported phenomenon in veterinary medicine, this study aimed to describe clinical and demographic factors in a large, prospectively identified cohort of dogs with T3‐L3 myelopathy with spinal shock and to compare those to a contemporaneous group of dogs with L4‐S3 myelopathy. We hypothesized that statistical models could be used to identify clinical variables associated with spinal shock in dogs with spinal cord injuries. This information could aid in diagnostic planning for dogs with thoracic and lumbar myelopathies.

## 2 METHODS

### 2.1 Animals and study design

Data used for this study were prospectively entered by partner institutions into the International Canine Spinal Cord Injury observational registry between October 2016 and July 2019. This database serves as a veterinary disease registry and aims to collect clinical data on dogs with spinal cord injury that are presented to specialty referral institutions. Details related to the database and its sponsor, CANSORT‐SCI, have been previously reported.^54^ Data for all dogs with a confirmed T3‐L3 or L4‐S3 myelopathies were originally extracted from the database. Dogs were assigned to 1 of 2 groups: those with clinical signs and imaging findings consistent with a T3‐L3 myelopathy with spinal shock, and those with findings suggestive of an L4‐S3 myelopathy. Dogs were included in the “T3‐L3 with spinal shock” group if they had lesions at or cranial to the L2‐L3 disc space and no other structural lesions were identified that could explain 1 or more: reduced pelvic limb reflexes, reduced pelvic limb tone, reduced perineal reflex, or decreased perianal tone, were included in the spinal shock group. Dogs with lesions at or caudal to the L3‐L4 disc space were included in the L4‐S3 group. Demographic and clinical data elements prospectively collected by the CANSORT‐SCI database are summarized in Table 1.^54^

**TABLE 1:** Demographic and clinical data elements prospectively collected by the CANSORT‐SCI database

Data collected
Breed
Sex (MN, MI, FS, FI)
Age (years)
Weight (kg)
Body condition score (1‐5/5)
Duration of clinical signs (hours)
Modified Frankel score (0‐5)^a^
Injury site
Diagnosis
Cutaneous trunci cutoff (yes/no)
Schiff‐Sherrington posture (yes/no)
Patellar reflex (normal, decreased/absent)
Pelvic limb withdrawal (normal, decreased/absent)
Pelvic limb tone (normal, decreased/absent)
Perianal reflex (normal, decreased/absent)
Perianal tone (normal, decreased/absent)

Abbreviations: FI, female intact; FS, female spayed; MI, male intact; MN, male neutered.

^a^ Modified Frankel score used by CANSORT‐SCI defines a score of 0 as paraplegia with absent superficial and deep pain sensation and a score of 5 as paraspinal hyperesthesia only.

### 2.2 Statistical analysis

Univariable logistic regression analyses were performed to assess significance of breed, sex, age, weight, body condition score, duration of clinical signs, injury severity, cutaneous trunci cutoff, Schiff‐Sherrington posture, patellar reflex, pelvic limb withdrawal, and pelvic limb tone on the odds of spinal shock. Independent variables were included in the initial multivariable logistic regression model if they had a significant effect (*P* ≤ .1) on the odds of spinal shock in univariable logistic regression.

**FIGURE 1:**
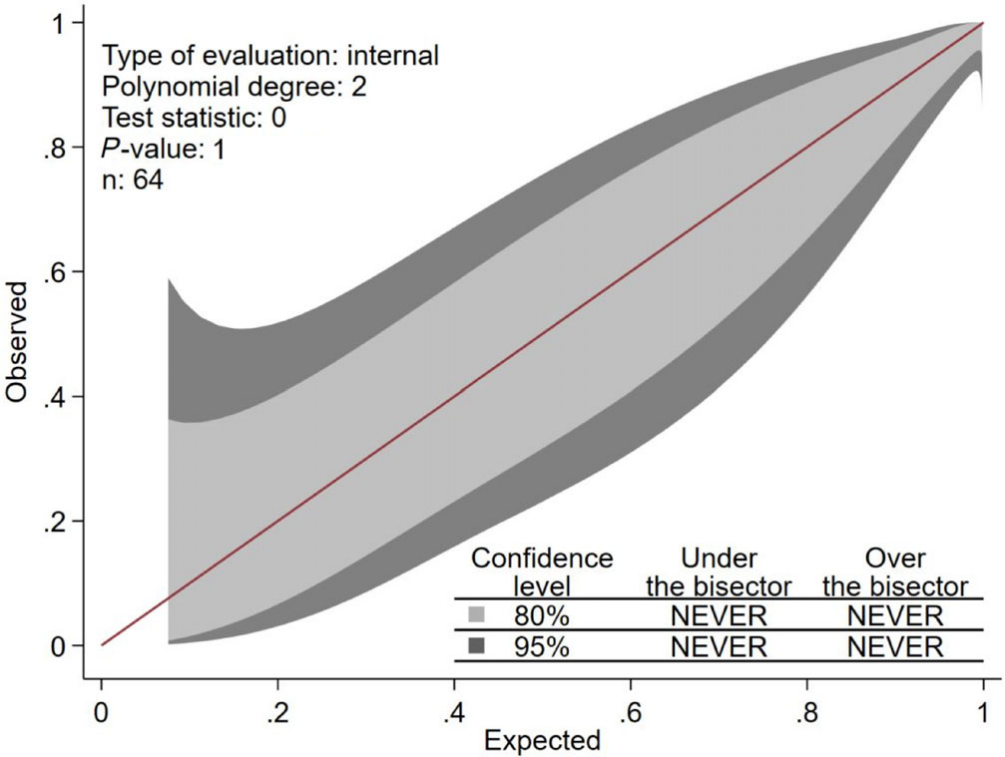
Calibration belt curve used for internal validation of the model used to predict the presence of spinal shock in dogs

A multivariable logistic regression model was then constructed to compare the odds of spinal shock in dogs with decreased pelvic limb reflexes or tone, presenting with different neurological examination findings, adjusting for the weight of the dog and the duration of clinical signs. Only dogs with complete datasets were included in the multivariable analysis. A backwards selection process was used to test each independent variable for inclusion in the final model with criteria of *P* ≤ .05 to remain. The best fitting model with the lowest Akaike information criterion (AIC) was selected and the Hosmer‐Lemeshow test was used to assess the fit of the model. Internal validation of the fitted model, including calibration and discrimination, was assessed using calibration belt (Figure 1) and the Area under the receiver operating characteristic (ROC) Curve (Supporting Information). The probabilities of spinal shock for dogs with different presentation scenarios were then calculated using postestimation marginal analyses. All data analyses were completed using StataCorp software (StataCorp Software, version 13; StataCorp LP, College Station, Texas).

## 3 RESULTS

### 3.1 Animals

**FIGURE 2:**
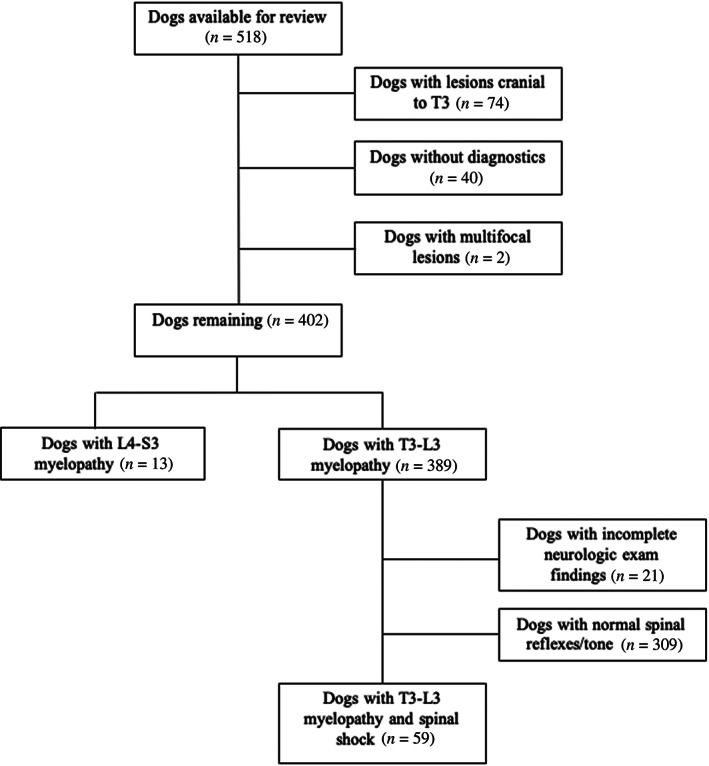
Flowchart documenting the numbers of dogs available from the CANSORT database and exclusion criteria applied to identify the T3‐L3 myelopathy with spinal shock and L4‐S3 myelopathy groups included in the present study

A total of 518 dogs with spinal cord injury were available in the database for evaluation. Once exclusion criteria were applied, data from 72 dogs remained for inclusion in the present study; 59 dogs with T3‐L3 myelopathy and spinal shock and 13 dogs with L4‐S3 myelopathy (Figure 2). Demographic and clinical features of both groups are summarized in Table 2.

**TABLE 2:** Demographic information for dogs with T3‐L3 myelopathies and spinal shock group and the L4‐S3 myelopathies available for inclusion from the CANSORT‐SCI database

	T3‐L3 myelopathy with spinal shock		L4‐S3 myelopathy	
	59		13	
Number of dogs (n)	MN (22), MI (6), FS (28), FI (3)		MN (7), MI (2), FS (4)	
Sex	Median	Range	Median	Range
Age (years)	5	(2‐13)	5	(2‐10)
Weight (kg)	10	(4‐50)	16	(8‐41)
Body condition score	3	(2‐5)	4	(3‐4)
Duration of clinical signs (h)	24	(1‐1080)	48	(3‐720)

Abbreviations: FI, female intact; FS, female spayed; MI, male intact; MN, male neutered.

### 3.2 Clinical findings

Median duration of clinical signs in the T3‐L3 with spinal shock group was 24 hours (range, 1‐1080 hours). Injury severity included ambulatory paraparesis (4), nonambulatory paraparesis (17), paraplegia with intact nociception (14), paraplegia with absent superficial but intact deep pain sensation (2), paraplegia with absent deep pain sensation (22). A cutaneous trunci cutoff was present in 45/59 dogs. Clinical signs attributed to spinal shock were: decreased pelvic limb tone was observed in 14/59 dogs, decreased patellar reflexes were observed in 14/59 dogs, decreased withdrawal reflexes were observed in 45/59 dogs. Multiple pelvic limb signs of spinal shock were noted in 18/59 dogs. Schiff‐Sherrington posture was present in 8/59 dogs. Specific injury sites identified on advanced imaging included T8‐T9 (1), T9‐T10 (1), T10‐T11 (4), T11‐T12 (14), T12‐T13 (25), T13‐L1 (15), L1‐L2 (3), and L2‐L3 (7). Forty‐nine dogs had single‐site compression identified on imaging and 10 dogs had multisite compression identified. Diagnoses based on magnetic resonance imaging (MRI) findings included acute intervertebral disc herniation (53), acute noncompressive nucleus pulposus extrusion (ANNPE; 5), and fibrocartilaginous embolic myelopathy (FCEM; 1).

Median duration of clinical signs in the L4‐S3 myelopathy group was 48 hours (range, 3‐720 hours). Injury severity included ambulatory paraparesis (4), nonambulatory paraparesis (6), paraplegia with absent superficial but intact deep nociception (1), and paraplegia with absent deep nociception (2). A cutaneous trunci cutoff was present in 4/13 dogs. Decreased pelvic limb tone was observed in 8/11 dogs, decreased patellar reflexes were observed in 7/11 dogs, decreased withdrawal reflexes were observed in 8/11 dogs. Multiple pelvic limb signs of an L4‐S3 myelopathy were observed in 8/11 dogs. Schiff‐Sherrington posture was present in 4/13 dogs. Specific injury sites identified via MRI or computed tomography (CT) included L3‐L4 (3), L4‐L5 (9), L5‐L6 (3), L6‐L7 (2), and L7‐S1 (1). Nine dogs had 1 injury site and 4 dogs had multiple injury sites. Diagnosis in all dogs with L4‐S3 myelopathy was acute intervertebral disc herniation.

### 3.3 Statistical analysis

#### 3.3.1 Univariable analysis

Results of univariable analysis comparing clinical factors between the T3‐L3 and the L4‐S3 myelopathy groups and their relationship to the presence of spinal shock are summarized in Table 3. Variables identified as significant (*P* ≤ .1) during univariable analysis, and therefore carried forward for multivariable analysis included: Sex (male vs female), breed category, natural log of weight in kg (Log_e_ weight) and the natural log of duration of clinical signs in hours (Log_e_ duration), severity of injury (paraparesis vs paraplegia), presence of a cutaneous trunci cutoff, decreased pelvic limb tone, and decreased patellar reflex. Decreased pelvic limb withdrawal, decreased perineal reflex, decreased anal tone, and Schiff‐Sherrington posture were not significant predictors of spinal shock in our study sample, based on our threshold for significance.

**TABLE 3:** Summary of univariable logistic regression analysis of continuous and binary variables

Predictor	N	OR SS	OR CI SS	OR L4‐S3	OR CI L4‐S3	*P*‐value
Log_e_ weight (kg)	69	0.36	0.14‐0.99	2.76	1.04‐7.3	.04
Log_e_ duration (h)	69	0.55	0.32‐0.92	1.82	1.08‐3.09	.02
Paresis = 0 Paraplegia = 1	72	6.03	1.5‐24.36	0.17	0.04‐0.67	.01
Presence of a cutaneous trunci cutoff	72	8.81	2.31‐33.57	0.11	0.03‐0.43	.001
Decreased pelvic limb tone	70	0.12	0.03‐0.5	8.57	2‐36.77	.004
Decreased patellar reflex	70	0.17	0.05‐0.7	5.63	1.43‐22.08	.01
Sex (reference = male)	72	3.69	0.92‐14.78	0.27	0.07‐1.09	.06
Age	72	1.1	−0.14 to 0.33	0.9	0.72‐1.15	.42
Decreased pelvic limb withdrawal	70	1.21	0.28‐5.17	0.83	0.19‐3.56	.8
Decreased perineal reflex	70	0.33	0.05‐2.06	3.06	0.49‐19.2	.23
Decreased anal tone	70	0.71	0.13‐3.88	1.42	0.26‐7.78	.69
Presence of Schiff‐Sherrington posture	72	0.35	0.09‐1.42	2.83	0.7‐11.32	.35

*Note*: *P*‐values generated from the univariable analysis to evaluate significance on the odds of spinal shock are shown. Data with *P*‐values <.1 were selected for the multivariable analysis.

Abbreviations: CE, coefficient; CI, confidence interval; OR, odds ratio; PL, pelvic limb; SS, spinal shock.

#### 3.3.2 Multivariable analysis

The best fitting model with the smallest AIC included Log_e_ weight, Log_e_ duration, severity score and pelvic limb tone (Table 4). There was no significant difference between the observed and the model expected values (Hosmer‐Lemeshow goodness of fit test, chi squared = 0.7).

**TABLE 4:** Final multivariable logistic regression model for T3‐L3 myelopathy with spinal shock with the smallest AIC included the natural log of weight, natural log of duration of clinical signs, pelvic limb tone, and, paresis vs paraplegia

Variable	Coefficient	Odds ratio (OR)	OR *P*‐value	OR confidence interval
Log_e_ weight (kg)	−1.27	0.28	.09	0.07‐1.2
Log_e_ duration (h)	−0.82	0.44	.02	0.21‐0.9
Paraplegia	2.06	7.87	.04	1.1‐56.62
Decreased pelvic limb tone	−3.11	0.04	.003	0.01‐0.36

**FIGURE 3:**
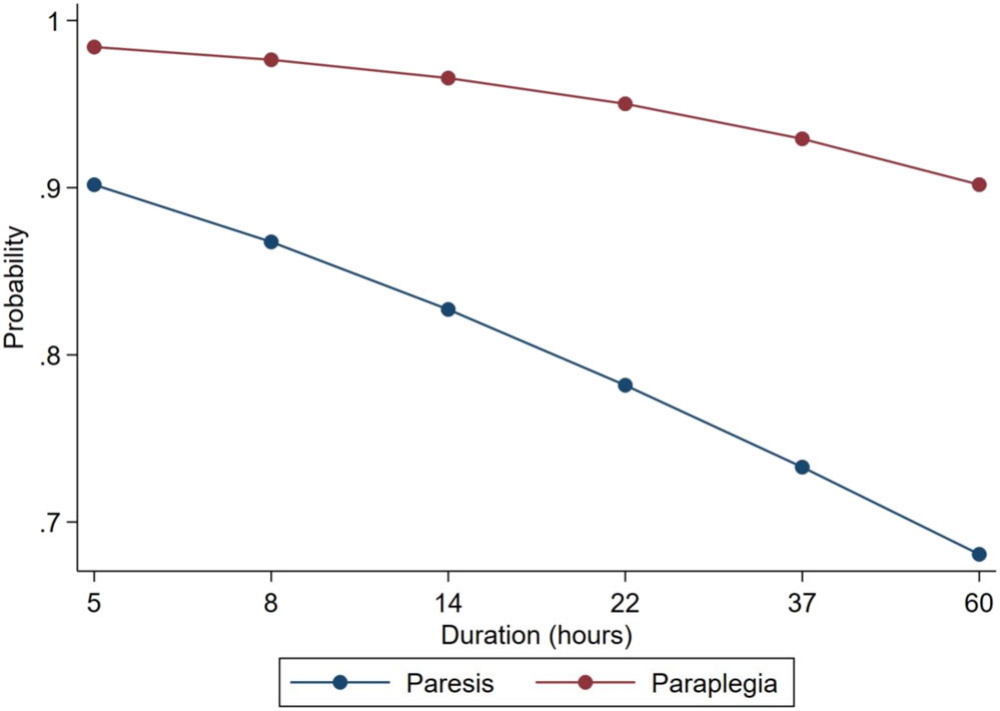
Graph demonstrating the relationship between duration of clinical signs in hours, paraparesis, and the probability of spinal shock in a 10 kg dog. With increasing duration of clinical signs and the presence of paraparesis, the probability of spinal shock decreases

**FIGURE 4:**
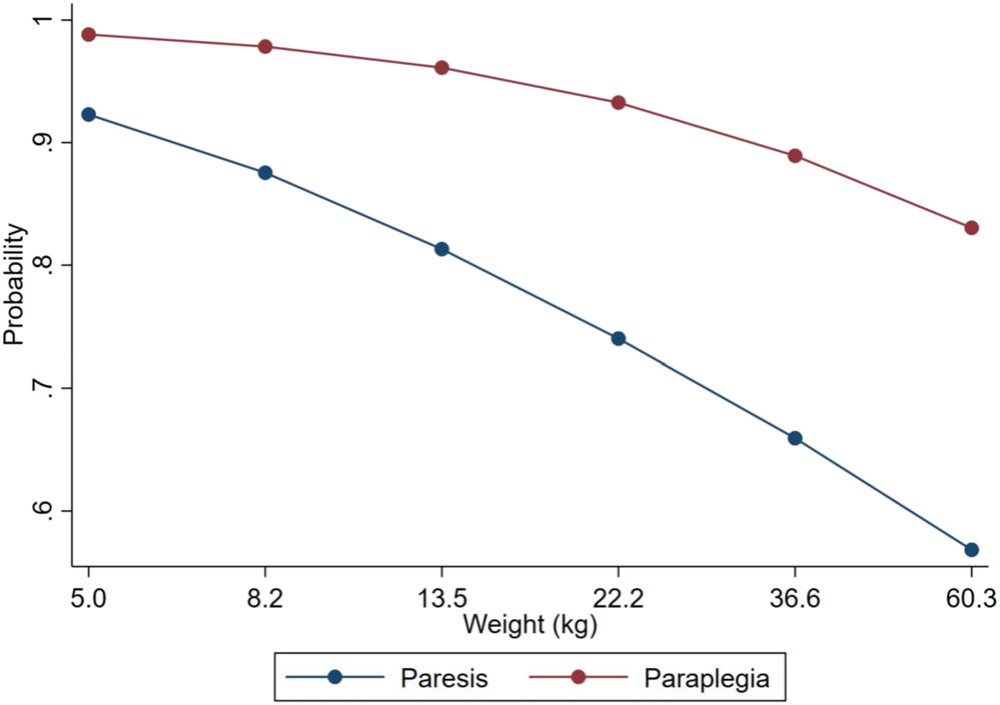
Graph demonstrating the relationship between weight in kilograms, paraparesis, and the probability of spinal shock for dogs that are presented at 10 hours after the onset of clinical signs. With increasing weight and the presence of paraparesis, the probability of spinal shock decreases

When other independent variables were kept constant, the odds of spinal shock increased with decreasing weight and decreasing duration of clinical signs (Figures 3 and 4). The odds of spinal shock in paraplegic dogs were 7.87 times greater than dogs with paraparesis. Dogs with decreased pelvic limb tone were 88% less likely to have a T3‐L3 myelopathy with spinal shock.

From the multivariable model, a formula was generated to calculate the odds of spinal shock using weight, duration of clinical signs, paraplegia (yes or no), and decreased pelvic limb tone (yes or no).

The formula for calculating the odds of spinal shock given certain predictors is:

$$e^{\beta_{o}+\beta_{1}\log_{e}\text{weight}+\beta_{2}\log_{e}\text{duration}+\beta_{3}\text{severity}+\beta_{4}\text{pelvic limb tone}}=e^{8.34-1.27*\log_{e}\text{weight}\;\left(\mathrm{kg}\right)-0.82*\log_{e}\text{duration}\;\left(\text{hours}\right)+2.06*\text{severity score}-3.11*\text{pelvic limb tone}}.$$

Severity score=1is paraplegic otherwise=0.

Pelvic limb tone=1if decreased/absent otherwise=0.

To illustrate common clinical presentations 2 scenarios were put into the model and are listed in Table 5. For example, Scenario 1 evaluates the probability of spinal shock in a 5 kg dog that is presented for an acute onset of paraplegia of less than 12 hours duration, and decreased pelvic limb tone. In that dog, the probability of spinal shock is 97%. In contrast, a 20 kg dog that is presented for ambulatory paraparesis and a history of clinical signs of greater than 48 hours duration, and decreased pelvic limb tone has a probability of spinal shock of 15%.

**TABLE 5:** Probability of spinal shock for different Log_e_ weight and Log_e_ duration for dogs that are presented with paraparesis vs paraplegia and decreased pelvic limb reflexes or tone

Dog weight in kg (Log_e_ weight)	Duration of clinical signs in hours (Log_e_ duration)	Paresis vs paraplegia	Decreased pelvic limb tone	Probability of spinal shock	95% confidence interval
5 (1.61)	11 (2.4)	Paraplegia	Yes	0.96	0.88‐1
20 (3)	49 (3.89)	Paraparesis	Yes	0.15	0‐0.36

## 4 DISCUSSION

Our study demonstrates that several key clinical factors can assist the clinician in prioritizing a T3‐L3 myelopathy with spinal shock over an L4‐S3 myelopathy to aid in diagnostic approaches. These include weight, duration of clinical signs, more severe neurologic grade (eg, the presence of paraplegia as compared to paraparesis), and the presence of decreased pelvic limb tone. In the present study, dogs with a lower body weight were more likely to have spinal shock than their larger counterparts. In addition, dogs with a duration of clinical signs <24 hours were more likely to have spinal shock. This is consistent with a case series of dogs with spinal shock, where 16/17 dogs had clinical signs of less than 24 hours duration.^4^ These might reflect differences in underlying causes of spinal cord injury between large and small breeds and might be specific to the sample included in our study. Small dogs, particularly those with chondrodystrophy, tend to be predisposed to acute episodes of intervertebral disc herniation that involve sudden rupture of the nucleus pulposus, often forceful expulsion of disc material into the vertebral canal, and associated rapid compressive and concussive injury to the spinal cord.^2^ Larger breeds are more prone to chronic, incrementally worsening intervertebral disc protrusions of the annulus fibrosus, which can present acutely when there is exacerbation of existing compression. The chronicity of this condition might allow the ascending and descending tracts associated with spinal shock to adapt accordingly. Previous experimental models have evaluated the role of chronicity in the development of spinal shock in cats by performing complete transections at the level of previously performed hemi‐sections in the thoracic spinal cord. In the cats with prior hemi‐sectioning, the presence and duration of spinal shock are attenuated.^7^, ^16^ Perhaps the formation of new pathways within the spinal cord that lead to both plasticity and hyperreflexia are already established with chronic lesions and allow for a quicker recovery from lack of cerebral input.^49^

Experimental models have also shown neurochemical alterations in the local reflex arc after spinal shock such as increased glycine uptake (inhibitory neurotransmitter), decreased serotonin uptake (excitatory neurotransmitter), reduction in voltage‐gated sodium and calcium persistent inward currents, and hyperpolarization of the resting membrane potential.^5^, ^24^, ^25^, ^26^, ^28^, ^31^, ^42^, ^44^ Perhaps with time, these neurochemical alterations can be reversed, potentially explaining the lack of spinal shock seen in animals with chronic myelopathies. It should be noted that the data collection instrument associated with the CANSORT‐SCI registry asks clinicians to choose a diagnosis based on MRI findings such as “acute intervertebral disc herniation”, “ANNPE,” or “FCE.” While the term herniation is often used broadly to encompass both extrusion of the nucleus pulpous and protrusion of the annulus fibrosus, inability to specifically differentiate the 2 disc‐related phenomena during data collection might explain this difference. While not specifically known from data included in the present study, it is possible that large breed dogs in our L4‐S3 myelopathy group could represent a previously documented distinct presentation encountered in veterinary medicine reflecting an acute on chronic presentation of annulus fibrosus protrusion.

Our results also demonstrated that paraplegic dogs had increased odds of spinal shock compared to those with persistent motor function. This likely relates to the fact that severity of neurologic signs often reflects the severity of spinal cord lesions.^2^, ^55^ In nonprimates, the predominant tracts involved in voluntary locomotion include the rubrospinal, reticulospinal, and vestibulospinal tracts.^1^, ^2^ These tracts start within the brainstem and descend in the spinal cord within the ventral and lateral funiculi.^1^, ^16^ Depending on the severity of the injury, lack of input from these tracts can result in paraparesis or paraplegia. Similarly, these tracts are postulated to provide input to the lower motor neurons of the pelvic limbs and their lack of input likely contributes to the development of spinal shock. Thus, a direct anatomic relationship between motor pathways and contributors to spinal shock might explain this finding.

The univariable analysis identified a strong association between the presence of a cutaneous trunci cutoff and a diagnosis of T3‐L3 myelopathy with spinal shock. The presence of a cutoff, however, was not independent of other variables, specifically duration of clinical signs. Therefore, the presence or absence of a cutaneous trunci cutoff in any given animal should be interpreted in the context of other clinical sings, including the dog's weight, and the duration and severity of clinical signs. The cutaneous trunci reflex starts with the cutaneous dorsal spinal nerves associated with each spinal cord segment. That information then ascends through the fasciculus proprius to C8‐T1 where it synapses on the cell bodies of the lateral thoracic nerve and causes a bilateral contraction of the cutaneous trunci muscle.^1^ Any lesion from T2‐L4 can cause a cutaneous trunci cutoff. Therefore, if a cutaneous trunci cutoff is present, the lesion has a high likelihood of being within the T3‐L3 spinal cord segments. In our study, 45/59 (76.3%) of dogs with a T3‐L3 myelopathy and spinal shock had a cutaneous trunci cutoff and 4/13 (30.7%) of dogs with an L4‐S3 myelopathy had a cutaneous trunci cutoff. In the univariable analysis (looking only at the 1 predictor), dogs with a cutaneous trunci cutoff were 8.8 times more likely to have a T3‐L3 myelopathy with spinal shock than an L4‐S3 myelopathy. However, this clinical sign fell out of the multivariable model indicating that the other factors could explain the probability of T3‐L3 myelopathy with spinal shock without knowledge about the cutaneous trunci reflex. Cutaneous trunci cutoff did seem to be related to the duration of clinical signs at presentation, which was retained in the model. If a clinician is presented with a dog with a spinal cord injury and decreased pelvic limb reflexes or tone, and that dog has a cutaneous trunci cutoff, spinal shock should be strongly considered as the explanation for reduced pelvic limb reflexes unless the dog's weight, duration of clinical signs and paresis suggest otherwise.

Consistent with previous studies, our data demonstrated that a decreased withdrawal reflex is the most commonly reduced reflex noted in dogs with spinal shock (76%).^3^, ^4^ However, when comparing the spinal shock group to the L4‐S3 myelopathy group, decreased withdrawal reflexes were not significantly different between groups and therefore not a significant predictor of spinal shock based on our model. This might reflect the fact that a reduced withdrawal reflex was common in both groups, and therefore could not discriminate 1 specific lesion localization over another. As compared to a reduced withdrawal reflex, a diminished patellar reflex is a less common finding in dogs with spinal shock and was present in only 24% of dogs in our study.^3^, ^4^ This might be caused by the fact that the patellar reflex is a monosynaptic reflex and therefore less susceptible to local alterations along the synaptic cleft.

In the present study, decreased pelvic limb tone was observed in 14/59 (24%) of dogs with spinal shock and 8/11 dogs (73%) with L4‐S3 myelopathy, where dogs with decreased pelvic limb tone were 88% less likely to have a T3‐L3 myelopathy with spinal shock than a L4‐S3 myelopathy (*P* = .004). Several factors likely contribute to this finding. First, a lesion affecting the L4‐S3 spinal cord segments can directly affect the lower motor neurons that are contributing to 1 or more of the nerves associated with those spinal cord segments. These include the femoral, sciatic, and pudendal nerve. The femoral and sciatic nerve predominately innervate the extensor (quadriceps) and flexor muscles of the pelvic limb, respectively.^1^, ^2^ Both of these muscle groups are essential to maintain tone in order to execute a normal weight‐bearing stance. A dog with a L4‐S3 myelopathy might be more likely to have these lower motor neurons affected and therefore might be more likely to have decreased tone.

The presence of a Schiff‐Sherrington posture was not significant clinical factor associated with the presence of spinal shock in the present study. Schiff‐Sherrington posture can be seen in dogs with a spinal cord injury anywhere from T1‐L5, but is most commonly seen in dogs with a T3‐L3 myelopathy.^1^, ^2^ Four out of 13 dogs in the L4‐S3 myelopathy group showed signs of a Schiff‐Sherrington posture. The lesions in these dogs involved either the L3‐L4 or L4‐L5 intervertebral disc space, or both, supporting that this phenomenon can be seen in both T3‐L3 and L4‐S3 myelopathies; this might explain why Schiff‐Sherrington posture was not strongly associated with the presence spinal shock in our study sample.

In the present study, the most common diagnosis in dogs with spinal shock was an acute intervertebral disc herniation (90%), followed by an acute noncompressive nucleus pulposus extrusion (ANNPE; 8%), and fibrocartilaginous embolic myelopathy (FCEM; 2%). Acute intervertebral disc herniation (56%) is the most prevalent etiology in dogs with spinal shock, followed by FCE (33%), and ANNPE (11%).^3^ All dogs in the L4‐S3 myelopathy group were diagnosed with an acute intervertebral disc herniation. This is in contrast to a case series of dogs with spinal shock that found FCE to be the most prevalent (41%), followed by acute intervertebral disc herniation (35%) and ANNPE (24%).^4^ The high prevalence of acute intervertebral disc herniations in the present study might have been influenced by the origins of the CANSORT‐SCI database, which originally focused on collecting clinical data specifically from dogs with intervertebral disc herniation and was later expanded to capture information from dogs with other causes of spinal cord injury. Alternatively, it could simply reflect that acute intervertebral disc herniation is the most common cause of spinal cord injury in dogs.^56^

Limitations of the present study include data collected across multiple referral institutions across North America and Europe. While definitions are provided for data elements within the CANSORT‐SCI database, the potential for clinician‐dependent variability in interpretation of certain aspects of the neurologic exam such as pelvic limb tone, probably exist. Additionally, as previously mentioned, diagnostic assignments collected in the database do not distinguish between disc extrusion and disc protrusion, which could impact the clinical presentation of acute spinal cord injury observed within a given group. Furthermore, the database only collects information related to initial presentation, diagnosis, and acute treatment. Therefore, we are unable to report information related to longitudinal monitoring of spinal reflexes or other clinical findings throughout recovery. While outside the original aims of the study, serial monitoring of spinal reflexes over time after presentation could assist the clinician further in identifying spinal shock vs L4‐S3 myelopathy in dogs with compatible clinical signs. Lastly, further studies are required to assess the external validity of the developed model.

In conclusion, we identified several key clinical variables associated with spinal shock in dogs with spinal cord injuries. Specifically, in dogs presented with an acute onset of signs (<24 hours), a lower body weight (<10 kg), and paraplegia, reduced pelvic limb reflexes and tone are significantly more likely to be related to a T3‐L3 myelopathy with spinal shock rather than to the presence of an L4‐S3 myelopathy. These findings should aid clinicians diagnostic planning for dogs presented with signs of neurologic disease caused by spinal cord injury and reduced pelvic limb reflexes and withdrawals.

## CONFLICT OF INTEREST DECLARATION

Andrea Tipold serves as Associate Editor for the Journal of Veterinary Internal Medicine. She was not involved in review of this manuscript. No other authors have a conflict of interest.

## OFF‐LABEL ANTIMICROBIAL DECLARATION

Authors declare no off‐label use of antimicrobials.

## INSTITUTIONAL ANIMAL CARE AND USE COMMITTEE (IACUC) OR OTHER APPROVAL DECLARATION

Authors declare no IACUC or other approval was needed.

## HUMAN ETHICS APPROVAL DECLARATION

Authors declare human ethics approval was not needed for this study.

## Supporting information


**Figure S1**. Receiver operating characteristic (ROC) curve for internal validation of the model used to predict the presence of spinal shock in dogs.
