# Supplementary material for: Developing a predictive model for spinal shock in dogs with spinal cord injury
Source: J Vet Intern Med. 2022 Jan 10;36(2):663–71. doi: 10.1111/jvim.16352 (PMC8965241; doi:10.1111/jvim.16352)
Supplement: Supplementary file 1 — Figure S1. Receiver operating characteristic (ROC) curve for internal validation of the model used to predict the presence of spinal shock in dogs. [file JVIM-36-663-s001.pdf]

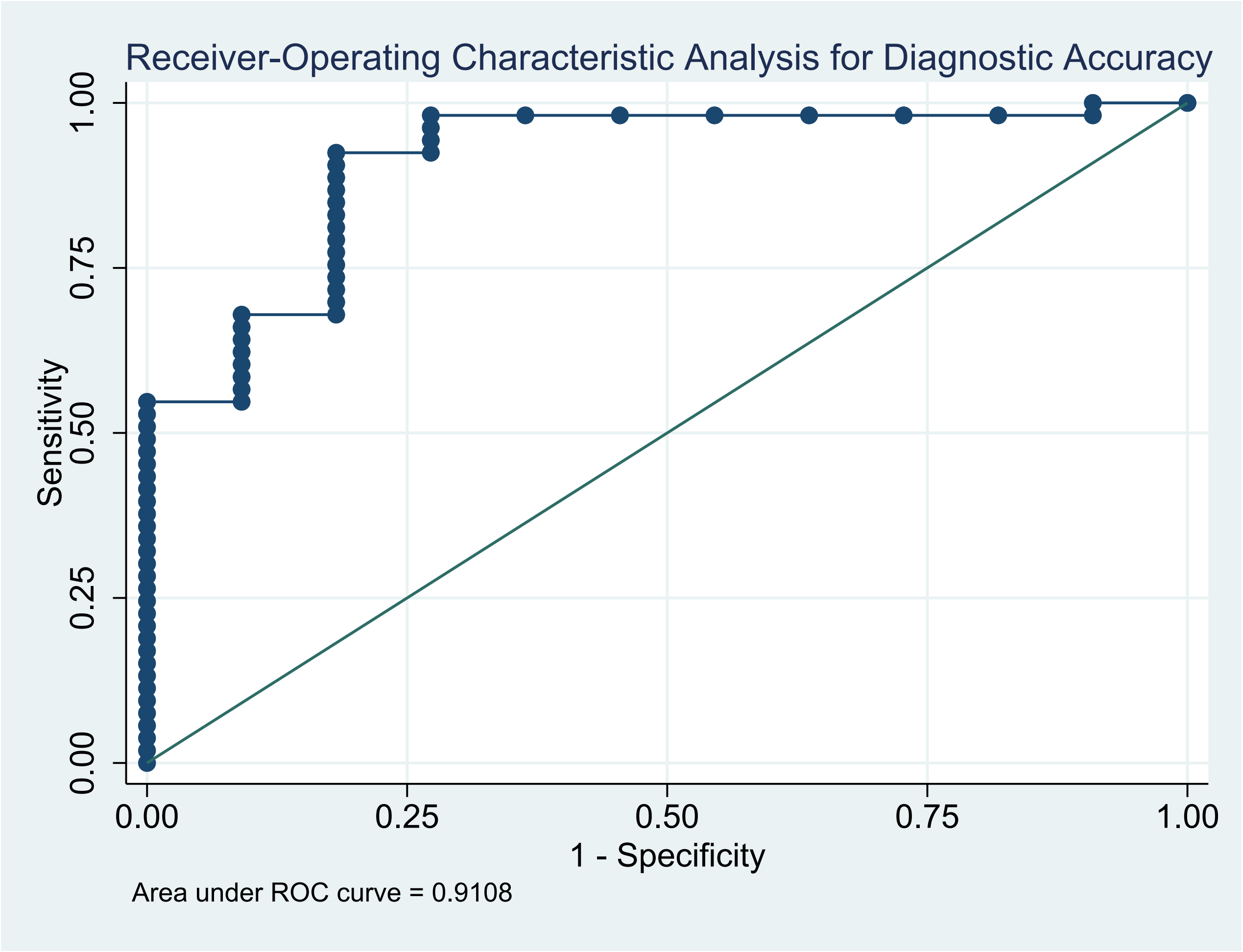

Supplementary Figure: Receiver Operating Characteristic (ROC) curve for internal validation of the model used to predict the presence of spinal shock in dogs
